# The Helicase Activity of Hyperthermophilic Archaeal MCM is Enhanced at High Temperatures by Lysine Methylation

**DOI:** 10.3389/fmicb.2015.01247

**Published:** 2015-11-09

**Authors:** Yisui Xia, Yanling Niu, Jiamin Cui, Yang Fu, Xiaojiang S. Chen, Huiqiang Lou, Qinhong Cao

**Affiliations:** ^1^State Key Laboratory of Agro-Biotechnology and Ministry of Agriculture Key Laboratory of Soil Microbiology, College of Biological Sciences, China Agricultural UniversityBeijing, China; ^2^Molecular and Computational Biology, Department of Biological Sciences, University of Southern California, Los AngelesCA, USA; ^3^USC Norris Comprehensive Cancer Center, University of Southern California, Los AngelesCA, USA; ^4^Department of Chemistry, University of Southern California, Los AngelesCA, USA

**Keywords:** protein methylation, DNA helicase, thermostability, hyperthermophiles, *Sulfolobus*

## Abstract

Lysine methylation and methyltransferases are widespread in the third domain of life, archaea. Nevertheless, the effects of methylation on archaeal proteins wait to be defined. Here, we report that recombinant sisMCM, an archaeal homolog of Mcm2-7 eukaryotic replicative helicase, is methylated by aKMT4 *in vitro*. Mono-methylation of these lysine residues occurs coincidently in the endogenous sisMCM protein purified from the hyperthermophilic *Sulfolobus islandicus* cells as indicated by mass spectra. The helicase activity of mini-chromosome maintenance (MCM) is stimulated by methylation, particularly at temperatures over 70°C. The methylated MCM shows optimal DNA unwinding activity after heat-treatment between 76 and 82°C, which correlates well with the typical growth temperatures of hyperthermophilic *Sulfolobus*. After methylation, the half life of MCM helicase is dramatically extended at 80°C. The methylated sites are located on the accessible protein surface, which might modulate the intra- and inter- molecular interactions through changing the hydrophobicity and surface charge. Furthermore, the methylation-mimic mutants of MCM show heat resistance helicase activity comparable to the methylated MCM. These data provide the biochemical evidence that posttranslational modifications such as methylation may enhance kinetic stability of proteins under the elevated growth temperatures of hyperthermophilic archaea.

## Introduction

Lysine methylation is one of the most common post-translational modifications, which can regulate the structure and function of protein. Remarkable progress has been made in the past decades in understanding how lysine methylation of histones regulates chromatin dynamics, gene expression, genome stability, and other cellular activities (Martin and Zhang, 2005). It has recently become evident that lysine methylation also occurs on non-histone proteins, suggesting its role far beyond epigenetics (Wu and Zhang, 2009; Erce et al., 2012; Zhang et al., 2012, 2015).

Besides chromatin proteins, many transcription factors were discovered to be methylated, such as p53, ERα, NFκB, E2F1, RB, TAF10, and STAT3 (Chuikov et al., 2004; Erce et al., 2012; Zhang et al., 2012; Clarke, 2013). The second class of non-histone proteins carrying lysine methylation belongs to translational apparatus, such as ribosomal proteins (e.g., Rpl1, Rpl23ab, Rpl42ab) and elongation factors (e.g., eEF1A, EF3A) in a diverse range of species from *Escherichia coli* to mammalians (Polevoda and Sherman, 2007). Furthermore, there are accumulating reports of lysine methylation of proteins involved in other cellular processes since its first discovery in a bacterial flagellar protein in (Ambler and Rees, 1959).

It is worth noting that lysine methylation may be prevalent in the third domain of life, Archaea (Choli et al., 1988; Febbraio et al., 2004; Botting et al., 2010). Abundant mono-methylation on lysine has been reported in *Sulfolobus* species, which usually thrive at hot springs throughout the world (Brock et al., 1972). For example, there is mono-methylation of 21 lysine residues in nine subunits of RNA polymerase complex purified from *Sulfolobus* (Botting et al., 2010). More recently, we and another group independently identified and characterized the first archaeal lysine methyltransferase, aKMT4, which may be, at least partially, responsible for variegated protein methylation in *Sulfolobus islandicus* (Chu et al., 2012; Niu et al., 2013). aKMT4, also named as ribosomal protein L11 methyltransferase, bears an eukaryotic KMT4/Dot1 family catalytic core and is well conserved throughout archaeal domain. In contrast to its distantly related homolog KMT4/Dot1 in eukaryotes, aKMT4 lacks substrate recognition domains which enables itself to target a set of nucleic acid metabolism related substrates including chromatin proteins (Sul7d, Cren7), RNA exosome subunits (Rrp4, Rrp42, and Csl4) and ribosomal proteins (Rpl11) *in vitro*. More strikingly, various lysine residues within the substrate can be mono-methylated to the different extent by aKMT4 due to multifaceted sequence specificity (Niu et al., 2013). Although several potential substrates have been identified, the effects of aKMT4 mediated protein methylation have yet to be defined.

Mini-chromosome maintenance (MCM) is an AAA^+^(ATPase with other associated cellular activities) superfamily protein harboring 3′–5′ DNA helicase activity, which is the replicative helicase and plays an essential role in initiation and elongation of DNA replication (Tye, 1999; Erzberger and Berger, 2006; Bochman and Schwacha, 2008, 2009; Sakakibara et al., 2009; Beattie and Bell, 2011; Onesti and MacNeill, 2013; Kelman and Kelman, 2014). In eukaryotes, MCM complex is a heterohexamer consisting of six paralogous MCM monomers (Mcm2-7). All six MCM subunits are essential for DNA replication and cell growth in yeast, while most of Archaea, including *Sulfolobales*, contain only one MCM subunit forming a homohexameric complex (Kelman et al., 1999; Chong et al., 2000; Fletcher et al., 2003; Barry and Bell, 2006; Bochman and Schwacha, 2009). Therefore, studies on the archaeal MCMs have been providing valuable insights into understanding the structure and function of eukaryotic Mcm2-7 complex (Fletcher et al., 2003, 2005; Barry and Bell, 2006; Moreau et al., 2007; Brewster et al., 2008; Slaymaker and Chen, 2012). Like its eukaryotic counterparts, MCM from *Sulfolobus* shows robust ATPase activity and 3′–5′ helicase activity *in vitro* (Pucci et al., 2004; McGeoch et al., 2005; Brewster et al., 2008). Several MCM subunits were shown to be phosphorylated by multiple kinases in yeast, which are proposed to be important in helicase activation during replication initiation step in yeast (Sheu and Stillman, 2006; Randell et al., 2010). Till now, there is still no direct evidence to show that MCM helicase activity can be modulated by post-translational modifications.

Here, we report that sisMCM can be mono-methylated at several lysine residues by aKMT4, and methylation can help to maintain higher helicase activity after treatment with elevated temperatures similar to that of the natural growth conditions of *S. islandicus*. Additionally, several methylation-mimic mutants of sisMCM behave similarly as the methylated MCM in maintaining helicase activity after heat treatment. These results provide experimental evidence that methylation contributes to protein thermostability and function in high temperatures.

## Results

### sisMCM is a Novel Substrate of aKMT4 *in Vitro*

**FIGURE 1:**
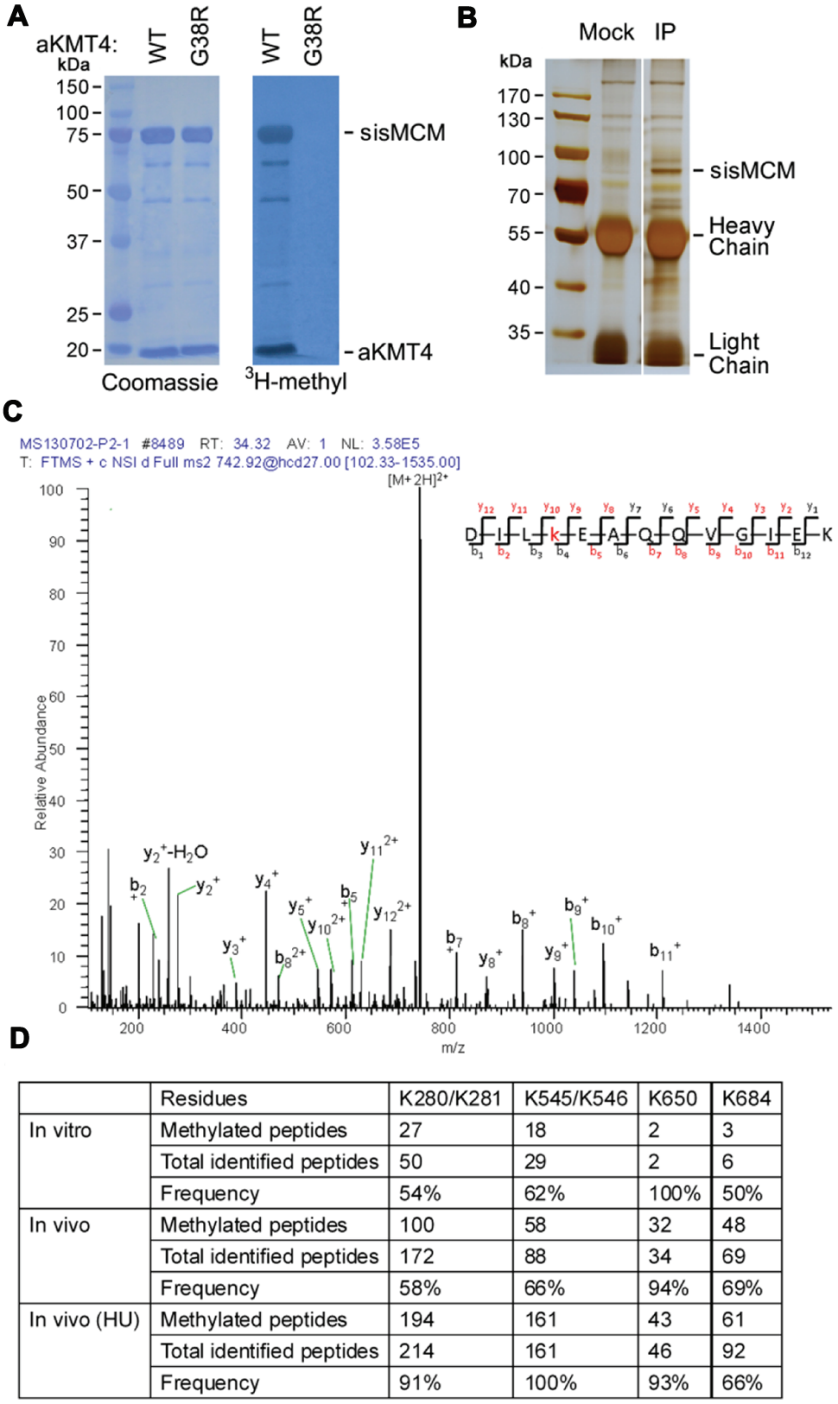
**sisMCM is mono-methylated on several lysine residues by aKMT4 *in vitro* and *in vivo*. (A)** Purified mini-chromosome maintenance (MCM) protein (2 μM) was incubated with the increasing amounts of either wild type aKMT4 or aKMT4-G38R mutant supplied with [^3^H-methyl]-AdoMet as a methyl donor in a total volume of 30 μL at 50°C for 3 h. The molar ratio of substrate to enzyme is 1:3. Proteins were separated by 10% SDS-PAGE for 2 h and transferred to a PVDF membrane. ^3^H-methyl incorporation was visualized by autoradiography. The membrane was also stained with Coomassie, which shows that MCM and aKMT4 migrate with apparent masses of 75 and 18 kDa, respectively. Note that MCM was methylated by aKMT4 but not by G38R mutant enzyme. **(B)** Immunoprecipitation of endogenous MCM protein from cell extracts of *Sulfolobus islandicus*. Native MCM protein was immunoprecipitated from lysates of *Sulfolobus* cells by anti-MCM antibody. A parallel immunoprecipitation was carried out as control using the mock antibody. **(C)** Multiple lysine residues in MCM protein were identified by MS/MS to be mono-methylated *in vitro* and *in vivo*. Recombinant MCM protein was methylated by aKMT4 under the standard methylation reaction *in vitro*. The methylated MCM, together with immunoprecipitated native MCM, were separated by 10% SDS-PAGE. Both MCM bands were sliced and in gel digested with trypsin prior to analysis by an Obitrap mass spectrometer. The collected MS and MS2 spectra were searched in NCBI protein database by MASCOT software. “b” and “y” ions with *m/z* difference by 14 may contain a mono-methylated lysine. A representative spectrum of MCM peptide containing K650me is shown. See Supplementary Figure S1 for spectra data of other sites. **(D)** The methylated lysine residues identified by MS/MS spectra. The hits of peptides carrying the putative methylation sites were tabulated. The methylation frequency is calculated by the percentage of the methylated peptides among total peptides containing the corresponding residues identified in MS/MS.

In a previous work, we identified the first conserved hyperthermostable lysine methyltransferase (aKMT4) in *S. islandicus*, which displays multifaceted substrate specificity (Chu et al., 2012; Niu et al., 2013). To explore whether there are other substrates of aKMT4, we cloned and expressed DNA polymerase and helicase genes from *S. islandicus*. Purified recombinant proteins were then subjected to the *in vitro* methylation reactions at 50°C for 3 h in the presence of [^3^H-methyl]-S-adenosyl-methionine (^3^H-AdoMet) as a methyl donor. Methylation by aKMT4 was detected by autoradiography of ^3^H-methyl incorporation. We noticed potent ^3^H-methyl incorporation into sisMCM protein as well as aKMT4 itself (**Figure 1A**). The automethylation of aKMT4 is consistent with our previous results (Niu et al., 2013). However, no tritium incorporation was observed in the presence of a methyltransferase-deficient mutant aKMT4-G38R, indicating sisMCM is specifically targeted by aKMT4. sisMCM is an archaeal homolog of eukaryotic DNA helicase Mcm2-7, which plays essential role in DNA replication initiation and progression (Fletcher et al., 2003; Barry and Bell, 2006). This indicates that DNA helicase sisMCM is methylated by aKMT4 *in vitro*.

### sisMCM is Mono-methylated *in Vivo* in a Similar Pattern as *in Vitro*

In order to examine whether MCM is methylated *in vivo* as well, we immunoprecipitated the endogenous MCM protein from *Sulfolobus* cells. First, we raised polyclonal anti-MCM antibody by immunizing rabbits with recombinant sisMCM purified from *E. coli*. Anti-MCM antibody was coupled to protein G beads. Purification was then carried out by adding cell extracts of the log phase *Sulfolobus* culture. After washed out non-specific associated proteins, the final bound fraction was boiled in SDS loading buffer and separated by 10% SDS-PAGE. Compared to mock beads, there was a significant enriched band in the anti-MCM precipitated fraction (**Figure 1B**). The enriched band was cut and in gel digested by trypsin. The tryptic fragments were subjected to an Orbitrap Velos. MS/MS spectra showed that endogenous MCM is indeed methylated at several lysine residues with different frequency *in vivo* (**Figures 1C,D** and Supplementary Figure S1). The variegated methylation pattern of MCM is in consistent with previous findings on other proteins in crenarchaea (Botting et al., 2010; Azkargorta et al., 2014) and degenerated sequence specificity of aKMT4 (Chu et al., 2012; Niu et al., 2013). To the best of our knowledge, this provides the first evidence that MCM family proteins can be modified by methylation.

Next, we compared the methylation pattern of MCM *in vivo* with that of *in vitro*. The *in vitro* methylation pattern was determined in a similar approach as described above using recombinant MCM catalyzed by aKMT4 in the standard methylation reaction. The endogenous methyllysine residues of MCM were detected to be coincidently targeted by aKMT4 *in vitro* to the similar extent (**Figure 1D**). It is noteworthy that these lysine residues (K280K281, K545K546, and K650) are located at the conserved regions of MCM homologs from crenarchaea, particularly in *Sulfolobables* genus (Supplementary Figure S2). Furthermore, only mono-methylation, but not di- or tri- methylation, was observed, which is consistent with the previous finding that aKMT4 is a non-processive enzyme (Niu et al., 2013). Given the similar methylation patterns between *in vitro* and *in vivo* conditions, aKMT4 is likely involved in MCM methylation in *Sulfolobus* though other possibilities cannot be ruled out in current stage.

### Methylation Stimulates the Helicase Activity of MCM at High Temperatures

**FIGURE 2:**
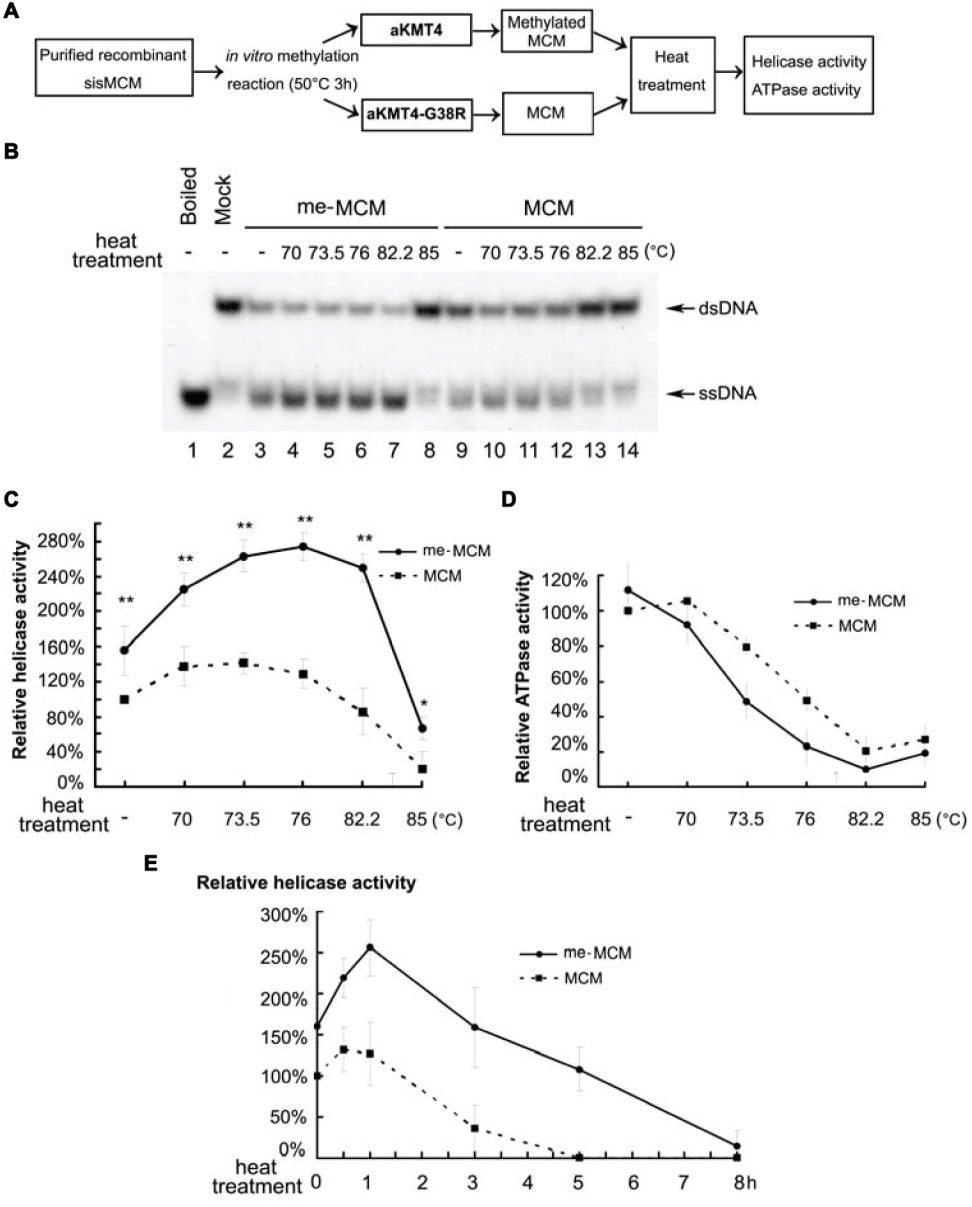
**Methylated MCM retains higher helicase activity after heat treatment. (A)** A general experimental flow chart for detecting the effect of methylation on thermostability and MCM functions. Same amounts of purified MCM protein were subjected to the *in vitro* methylation in the presence of wild type aKMT4 or aKMT4-G38R at 50°C for 3 h. aKMT4-G38R dead enzyme was applied as a control to ensure the un-methylated MCM protein undergoing the exactly same experimental procedure as the methylated MCM (me-MCM). Next, the reaction mixtures were subsequently heat-treated for 1 h at different temperatures between 70 and 85°C as indicated above each lane in **(B)**. The heat-treated samples were then analyzed for its helicase **(B,C)**, and ATPase **(D)** activities at 65°C. **(B)** Helicase activity after the heat-treatment of me-MCM and un-methylated MCM. me-MCM retained higher helicase activity after heat treatment compared to un-methylated MCM. The methylation and heat treatment were carried out as depicted in **(A)**. DNA products were separated by native PAGE gel. **(C)** Quantitation of the results in **(B)**. Error bars were calculated based on at least three independent experiments. ^∗∗^ and ^∗^indicate the significant difference by *p*-value < 0.01 and < 0.05, respectively. **(D)** ATPase activity after heat-treatment of the methylated and un-methylated MCM, showing that MCM methylation or not has mild effect on ATPase activity. **(E)** Helicase activity of me-MCM and MCM after heat-treatment at 80°C for the indicated times. The half lives of me-MCM and MCM under 80°C were calculated to be approximately 7.2 and 2.8 h, respectively.

Given the fact that *S. islandicus* cells grow optimally near 80°C (Brock et al., 1972; Contursi et al., 2006), we next reasoned that MCM methylation may contribute to the normal function of this key enzyme at such high temperatures. To address this possibility, we compared the modified MCM with the unmodified version for their catalytic activity. To minimize the artificial interference from *in vitro* manipulation, methylated (me-MCM) and un-methylated MCM were obtained through exact same methylation reactions at 50°C for 3 h in the presence of aKMT4 or inactive aKMT4-G38R methyltransferases, respectively (**Figure 2A**). Helicase assays were conducted at 65°C for 1 h using a ^32^P labeled fork-structured double stranded DNA (dsDNA) substrate. The unwound products were detected by autoradiography. The amounts of unwound single stranded DNA (ssDNA) to dsDNA were quantified by Quantity One (Bio-Rad). Neither aKMT4 nor aKMT4-G38R was contaminated with any helicase or ATPase activity (data not shown). There is an about 50% increase in DNA unwinding activity of me-MCM compared to that of un-methylated MCM (**Figures 2B,C**, compare lane 3–9). This result indicates that methylation causes a moderate stimulation on the helicase activity of MCM under the *in vitro* reaction conditions.

### Methylated MCM Displays Increased Heat Resistance

Such an effect could be underestimated because of the limitation of the *in vitro* reaction conditions. For example, to avoid melting of the fork-structured DNA substrates *in vitro*, the reaction temperature for helicase activity has to be kept much lower than the typical growth temperature of hyperthermophilic *Sulfolobus*. To address this issue, we examined the effect of heat treatment on helicase activity. Subsequent to *in vitro* methylation by aKMT4 (me-MCM) or aKMT4-G38R (MCM), samples were heated at the indicated temperatures for 1 h prior to the helicase activity assay (**Figure 2A**). Strikingly, compared with un-methylated one, the methylated enzyme produced more ssDNA from dsDNA substrates (**Figure 2B**, compare lanes 4–8 to 10–14). The quantitative analysis from three independent experiments showed the significant difference (*P*-value < 0.01) of the unwinding capacity between two versions of MCM (**Figure 2C**). me-MCM displayed around 1.5-fold higher helicase activity than MCM after heat treatment between 70 and 82°C (**Figure 2C**). The me-MCM reached the highest unwinding activity after heat-treatment at about 80°C, the typical temperature of the natural habitat of *Sulfolobus* (Zhang et al., 2015). On the other hand, ATPase activity of MCM was only slightly affected post methylation and heat treatment (**Figure 2D**). ATPase is a prerequisite of activity. However, in the previous structural and functional studies, MCM mutants bearing higher ATPase do not always show higher helicase activity (Barry et al., 2007; Moreau et al., 2007; Pucci et al., 2007; Jenkinson et al., 2009; Brewster et al., 2010). These data indicate that methylation can stimulate the helicase activity of MCM, and this effect became amplified above 70°C. The duration time to retain 50% of helicase activity was measured by heat treatment at 80°C for the indicated times. As shown in **Figure 2E**, the half lives of me-MCM and MCM at 80°C were estimated to be approximately 5.9 and 2.8 h, respectively. This result indicates that methylation can increase the half life of MCM helicase, i.e., kinetic stability, which may count for its normal function at temperatures over 70°C.

### Methylation Poses Subtle Effects on Thermodynamics of MCM

**FIGURE 3:**
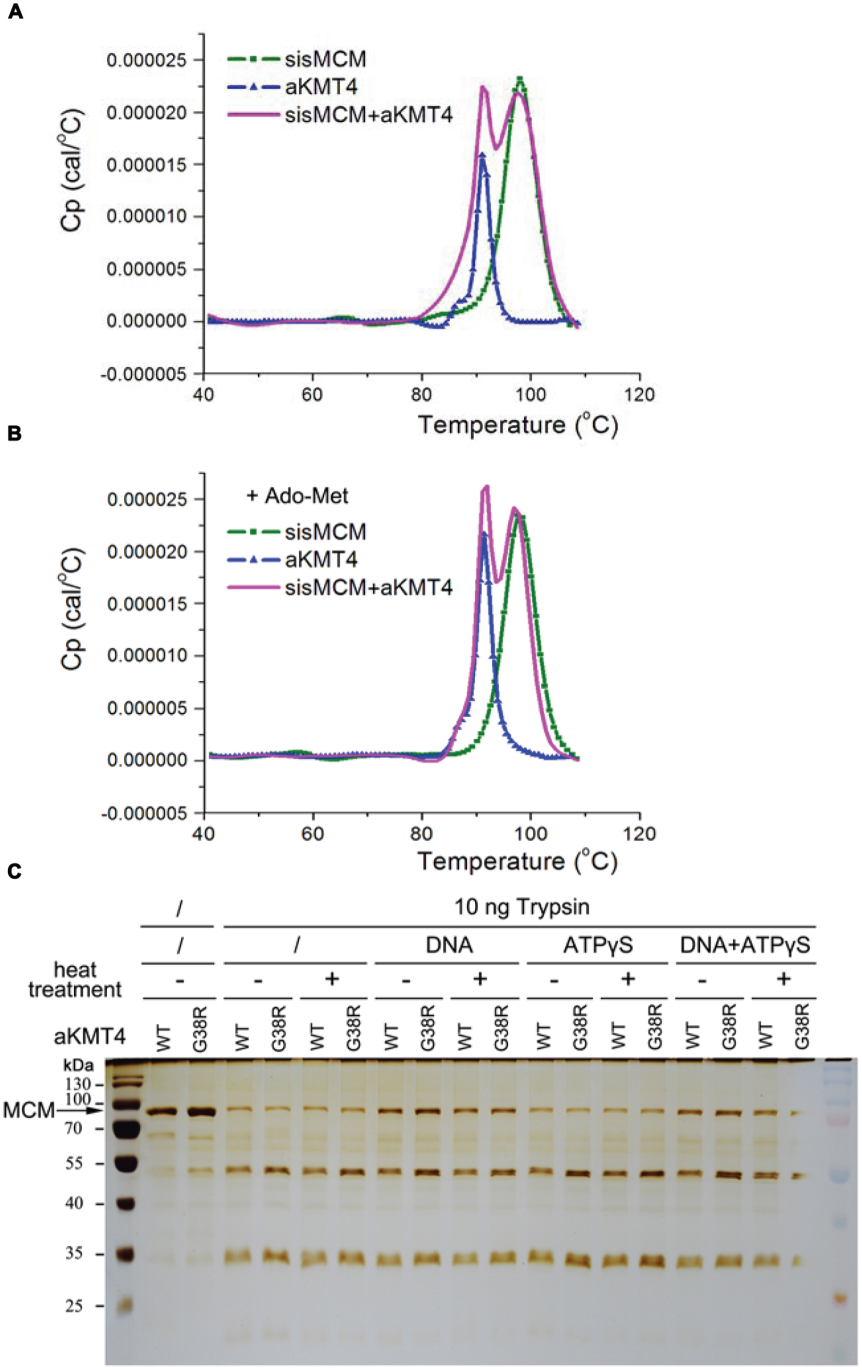
**Methylation shows only subtle effect on MCM conformation and thermodynamics stability. (A)** The melting curves of sisMCM, aKMT4 and their mixture in the absence of Ado-Met during thermal denaturation measured by differential scanning calorimetry (DSC). Purified MCM (2.6 μM) and aKMT4 (9.4 μM) were incubated at 50°C for 3 h in the standard methylation reactions. The samples were next scanned by DSC from 20 to 110°C with a rate of 2°C/min. Note that the mixed MCM and aKMT4 showed essentially identical *T*m values corresponding to each separated component, indicating two proteins do not interfere with their thermodynamics stability of each other. **(B)** The melting curves of sisMCM, aKMT4 and their mixture in the presence of Ado-Met during thermal denaturation measured by DSC. When Ado-Met was added to the methylation reactions, both MCM and aKMT4 can be methylated. However, there is no significant change of the melting curves compared to the ones in the absence of Ado-Met as shown in **(A)**. **(C)** The limited trypsin digestion analysis of the methylated and un-methylated MCM, showing that modified and unmodified MCM have similar digestion patterns. MCM proteins were treated as described in **Figure 2A** prior to incubation with 20 nM trypsin in the presence or absence of ATP analog at 25°C for 15 min.

We next determined whether methylation affects the MCM thermodynamic stability, another aspect of protein thermostability. Thermodynamic stability is defined by free energy of stabilization (Δ*G* stab) and by the melting temperature (*Tm*; Vieille and Zeikus, 2001). The enzymes often unfold irreversibly, therefore we measured *T*m (at which 50% of the enzyme is unfolded) by the differential scanning calorimetry as an indicator of dynamic stability. Each sample was loaded into a Nano-DSC II calorimeter and scanned from 20 to 110°C at a rate of 2°C/min. Recombinant sisMCM and aKMT4 alone have an apparent *T*m of 98 and 91°C, respectively (**Figure 3A** and **Table 1**). This result indicates the robust thermodynamic stability of these two proteins, while the relatively lower *T*m of aKMT4 is in agreement with its less thermostability *in vitro* (Niu et al., 2013). When MCM was incubated with aKMT4 at 50°C for 3 h in the absence of the methyl-donor Ado-Met, two peaks were observed at about 97.5 and 91.7°C, corresponding to the peaks of MCM and aKMT4, respectively (**Figure 3A** and **Table 1**). This pattern indicates that co-existence of MCM and aKMT4 does not interfere with thermodynamics of each other under the *in vitro* reaction conditions. When Ado-Met was added to trigger MCM methylation, the melting curves of both MCM and aKMT4 were kept almost unperturbed (**Figure 3B** and **Table 1**). This suggests that methylation does not change the melting temperature of MCM dramatically. It is worth noting that aKMT4 also showed similar melting pattern regardless of existence of Ado-Met. At least part of aKMT4 can be methylated as well in the presence of Ado-Met due to its unique self-methylation activity (**Figure 1A**; Niu et al., 2013). These suggest that there is no detectable dynamic change induced by MCM methylation.

**Table 1:** Thermodynamic parameters of aKMT4, mini-chromosome maintenance (MCM) and their methylated forms analyzed by differential scanning calorimetry (DSC).

MCM	aKMT4	Ado-Met	*T*_m_ of aKMT4 (°C)	*T*_m_ of MCM (°C)
+	-	-	-	97.6 ± 0.3
-	+	-	91.1 ± 0.2	-
+	+	-	91.4 ± 0.3	97.8 ± 0.3
+	-	+	-	97.4 ± 0.4
-	+	+	91.2 ± 0.2	-
+	+	+	91.6 ± 0.3	97.4 ± 0.3

*The error values are derived from the fitting of three independent experiments*.

To investigate whether methylation can induce conformational changes which may count for increased helicase activity after heat-treatment, we compared the limited tryptic digestion profiles of MCM with or without methylation. MCM became partially resistant to trypsin digestion after association with dsDNA, but not with ATPγS, a non-hydrolyzable analog of ATP (**Figure 3C**). This indicates that the conformational changes induced upon binding to dsDNA can be detected by the conditions used here. Nevertheless, me-MCM showed basically the same digestion pattern as the unmodified one regardless of whether they were heat treated or not (**Figure 3C**). Taken together, these data indicate that methylation *per se* does not trigger significant structural or thermodynamic changes which can be detected under the conditions used here.

### Methyl Groups are not Directly Targeted to Catalytic Domains of MCM

**FIGURE 4:**
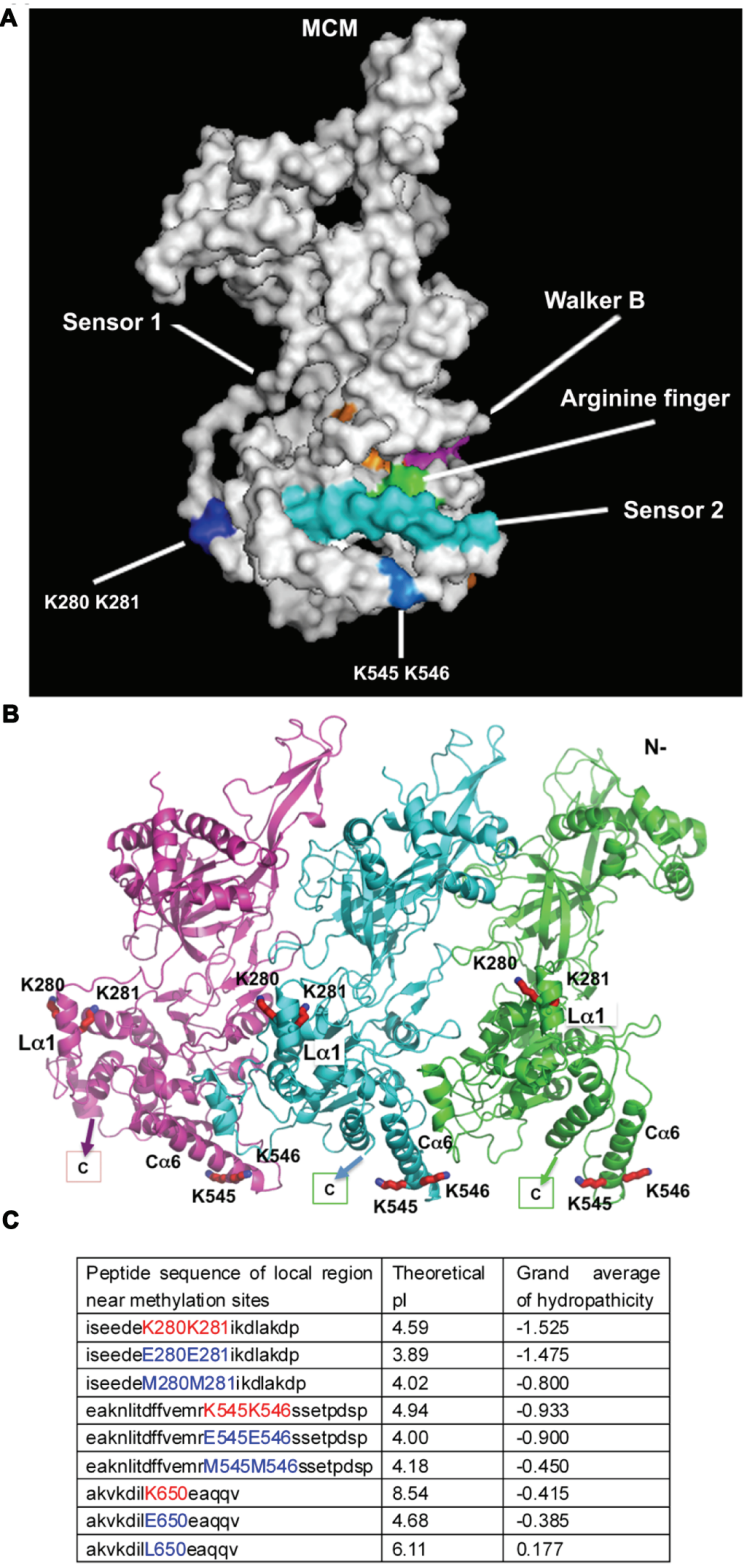
**Methyllysines are located on the accessible protein surface. (A)** Methylation sites are located on the protein surface away from the catalytic centers. Identified methylation lysine residues, labeled in blue, were mapped to the near full length three-dimensional structure of its highly homologous ssoMCM (based on PDB ID 4FDG). The conserved catalytic motifs are indicated according to (Brewster et al., 2008). **(B)** Mapping of the methylated lysine residues of sisMCM on to ssoMCM (PDB ID 4FDG) in the context of a hexameric ring/lock-washer conformation. For clarity only three subunits (in three discrete colors) in the front of a hexamer are shown. All four lysine residues found to be methylated (K280, K281, K545, and K546, shown in red sticks) are on the AAA+ helicase domain. K280 and K281 are located on the linker helix-Lα1 [helix nomenclature adapted from (Brewster et al., 2008)], and K545 and K546 on helix Cα6. They are all surface accessible. K546 is the only residue in bonding distance with a neighboring subunit, which may also change its rotamer conformation to interact with other protein partners. A small C-terminal winged helix domain (WHD; indicated) that should be in the neighborhood around K545 is absent from the structure. **(C)** Methylation simulation analysis. The peptide sequences nearby the methylated lysine were retrieved. Theoretical pI and grand average of hydropathicity of local area around methylation sites were calculated in ProtParam on the ExPASy Server (Wilkins et al., 1999). In order to mimic methylation, methyllysines were substituted by the amino acids carrying a longer side chain, like methionine or leucine. Glutamic acid substitution was applied to indicate the effect of charge change.

To gain insights into the role of MCM methylation, we examined the location of the methylation sites on the near full length *S. solfataricus* MCM structure in the context of a hexameric ring or lock-washer conformation (Brewster et al., 2008; Slaymaker et al., 2013). The four lysine residues subjected to mono-methylation (K280, K281, K545, K546) are located on the accessible protein surface in the C-terminal AAA+ helicase domain but away from the ATP catalytic pocket (**Figure 4A**). This likely provides a possible reason for why only helicase activity, but not ATPase, was significantly affected by methylation. Meanwhile, the location of these residues could explain why addition of a small methyl group to the side chain of these lysine residues did not lead to an obvious overall structural change. However, methylation will affect side chain conformation of a lysine by adding a hydrophobic methyl group. K280/K281 on the linker helix 1 (Lα1, **Figure 4B**) with around 4–5 Å distance to another helix of the AAA+ domain, a distance ready to make bonding interactions with slight adjustment of side-chain conformation. The linker and its helix Lα1 connect the N-terminal domain and C-terminal AAA+ domain, and is important for helicase activity (Erzberger and Berger, 2006; Jenkinson and Chong, 2006; Barry et al., 2007, 2009; Brewster et al., 2008; Slaymaker and Chen, 2012). Methylation of K280/K281 could impact the linker structural flexibility at elevated temperatures to display higher unwinding activity. For K545/K546, they are located on the C-terminal helix 6 (Cα6, **Figure 4B**). K546 is next to the missing C-terminal winged helix domain (WHD) that can regulate helicase activity, and K546 is in bonding distance to a helix from a neighboring subunit (**Figure 4B**). Methylation of these two residues could subtly affect the interactions with WHD and its neighbor to result in a higher helicase activity at elevated temperatures by affecting hexameric conformational switches needed for efficient unwinding. Furthermore, we computated the changes of the local surface charge and the hydrophobic parameters which have been shown to be induced by methylation (Febbraio et al., 2004; Egorova et al., 2010; Clarke, 2013). To this end, we retrieved the local peptide sequence surrounding the methylated sites. The theoretical pI and grand average of hydropathicity were calculated (**Figure 4C**). Next, the mono-methylated lysine residues were simulated by amino acids with a longer side chain, methionine (M) or leucine (L). The mimic mutants, such as MCM-K280M/K281M, MCM-K545M/K546M, and MCM-K650L, showed significant increased surface hydrophobicity and *pK*_a_ (**Figure 4C**), which may count for the enhanced overall thermostability.

### Methylation-mimic Mutants of MCM Show Heat Resistance as Well

**FIGURE 5:**
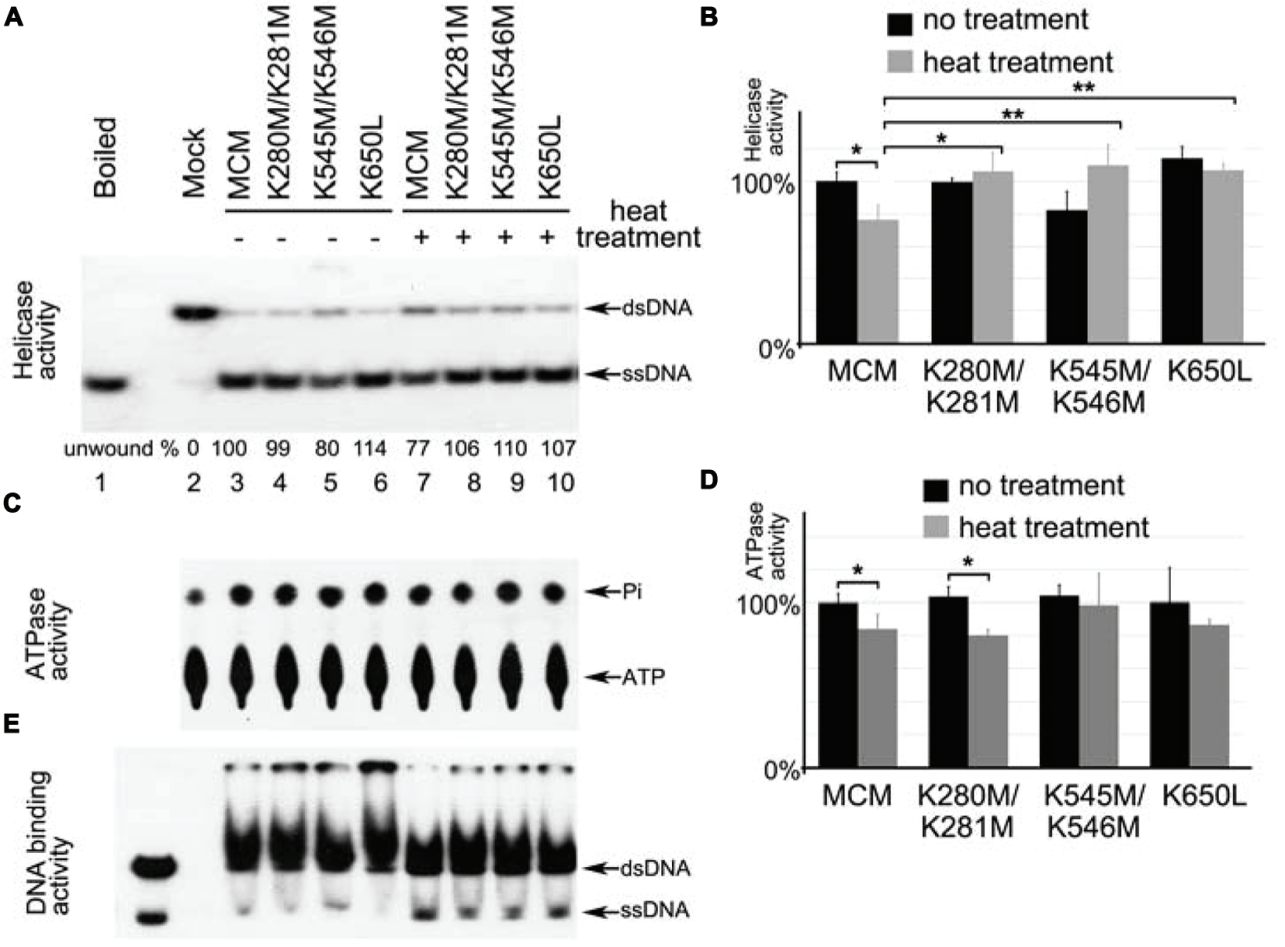
**Methylation-mimic MCM proteins show increased resistance to heat treatment**. Wild type and mutant MCM proteins were treated at 80°C for 1 h prior to measurement of their helicase activity **(A,B)**, ATPase activity **(C,D)** and DNA binding activity **(E)**. **(A,B)** All three MCM methylation-mimic mutants retain higher helicase activity after heat treatment than wild type MCM. **(C,D)** After heat treatment, MCM-K280M/K281M and wild type MCM show slightly decreased ATPase activity, while MCM-K545M/K546M and MCM-K650L retain their ATPase. **(E)** All methylation-mimic mutants showed no apparent change on their DNA binding after heat treatment. The DNA binding capability of MCM was measured by gel shift using ^32^P labeled fork-structured DNA. Significant differences between different samples are denoted by ^∗∗^*P*-value < 0.01 or ^∗^*P*-value < 0.05. All quantitative results are calculated from triplicates.

Next, we directly tested if the methylation-mimic mutants have similar performance as the methylated MCM at high temperatures as shown in **Figure 2**. The mutant proteins (MCM-K280M/K281M, MCM-K545M/K546M, and MCM-K650L) were expressed and purified for helicase assay. Without heat treatment, these MCM mutants showed comparable helicase activity to wild type (**Figures 5A,B**). After heat treatment at 80°C for 1 h, wild type MCM showed compromised helicase activity (**Figure 5A**, compare lane 4–8). However, with the same heat treatment, no reduction of unwinding was observed for all methylation-mimic mutants (**Figure 5A**, compare lanes 5–7 to 9–11). Thus, all methylation-mimic mutants retained significantly higher DNA unwinding activity than the unmodified enzyme (**Figure 5B**). As for ATPase, K280M/K281M showed moderately decreased activity as wild type, whereas no dramatic effect was observed for K545M/K546M and K650L mutants (**Figures 5C,D**). Gel shift assays showed no apparent changes for their binding to DNA substrates after heat treatment (**Figure 5E**). These results further support that methylation of MCM may improve its helicase activity *in vitro* at 80°C, a typical growth temperature of *Sulfolobus*.

## Discussions

Hyperthermophiles are valuable sources of thermostable enzymes displaying remarkable stability against high temperatures. Multiple mechanisms have been inferred in protein thermostability including hydrogen bonds, hydrophobicity, ion pairs, salt bridges, compactness, and surface charges (Vieille and Zeikus, 2001). In this study, besides these inherent intrinsic properties of protein, we provide biochemical evidence to support that methylation contributes to protein thermostability and functionality at temperatures over 75°C.

Most proteins from hyperthermophiles remain thermostable when they are expressed in mesophilic hosts such as *E. coli*. However, it’s not uncommon that recombinant proteins display relative weaker thermal resistance than their native counterparts (Chu et al., 2012; Niu et al., 2013). The optimal temperature of recombinant MCM enzyme is about 70°C, which is elevated to the typical growth temperatures of hyperthermophilic *S. islandicus* after methylation by aKMT4. More intriguingly, like methylated MCM, the recombinant methylation-mimic mutants display increased heat resistance than the unmodified enzyme. All these results support that methylation can enhance protein thermal properties.

Thermodynamic and kinetic stabilities represent two aspects of protein thermostability (Vieille and Zeikus, 2001). The melting temperature (*T*m) of MCM is not changed in the presence or absence of methylation, indicating methylation does not increase detectable thermodynamic stability. On the other hand, kinetic stability of MCM is potently enhanced as evidenced by the extended half life of MCM upon methylation. These findings suggest a novel regulatory role of methylation *per se* for maintaining MCM function at high temperatures in hyperthermophilic archaea, likely through increasing their kinetic stability rather than thermodynamic stability. Both aKMT4 and its targeted lysine residues in MCM are conserved in crenarchaea (Supplementary Figure S2; Chu et al., 2012; Niu et al., 2013). Moreover, native β-glycosidase in *S. solfataricus* bears up to five methyllysines, which shows resistance to heat denaturation and aggregation compared to the unmodified recombinant counterpart, particularly at high temperatures (Febbraio et al., 2004). Therefore, the thermo-adaptation strategy through protein methylation may be widely utilized among hyperthermophiles. The regulatory role of protein methylation identified here is in agreement with the increased lysine methylation of Sul7d observed at the elevated growth temperatures, which is methylated at multiple sites by aKMT4 as well (Choli et al., 1988; Febbraio et al., 2004; Botting et al., 2010; Chu et al., 2012; Niu et al., 2013). Genetic evidence will be needed in future to elucidate the physiological function of numerous methyltransferases like aKMT4 and their targets *in vivo*.

Methylation on protein surface should not cause major conformational change in protein structure. Nevertheless, it can provide a novel interface for association with other molecules, thus affecting numerous features of the methylated protein, including protein-protein interactions, localization, enzyme activity, and turn over. Through *in vitro* biochemical assays in the absence of other proteins, we are able to reveal the effect of lysine methylation *per se* on the helicase activity of MCM, presumably through altering free-energy for the local secondary structural movement within its homo-hexameric subunits at the elevated temperatures. The stimulation effect of methylation on helicase activity is significantly enhanced when the temperatures are elevated above 70°C. In mesophilic organisms, it has been proposed that methylation can improve protein solubility and stability (Egorova et al., 2010). Non-enzymatic methylation has being extensively used to improve protein crystallization but does not generally change the structure and activity of enzymes (Kim et al., 2008). Furthermore, methylation of amyloid peptide β-(25–35) increases the solubility, thus prevents the aggregation propensity, which is believed to cause Alzheimer’s disease (Hughes et al., 2000). Taken all together, it will be of interest to expand the effects of lysine methylation other than mediating protein-protein interactions to a broader scope.

## Experimental Procedures

### Organisms and Culture Conditions

*Sulfolobus islandicus* REY15A strain, a gift of Dr. Qunxin She at University of Copenhagen, was cultured at 75°C as described earlier (Contursi et al., 2006; Zhang et al., 2015).

### Gene Cloning and Mutagenesis

The coding sequence for sisMCM (*SiRe-1228*) was amplified using *S. islandicus* REY15A genomic DNA and cloned into pGEX-6P-1 (GE Healthcare) via NovoRec PCR (Novoprotein). Point mutations were introduced using the Quick Change site-directed mutagenesis kit from Stratagene. Mutations were confirmed by sequencing the entire coding region. Oligonucleotides were synthesized by Sangon Biotech.

### Protein Purification

All recombinant proteins were overexpressed in *E. coli* BL21 (DE3) CodonPlus RIL (Stratagene). GST-sisMCM and its mutant proteins were purified by glutathione-Sepharose columns (GE Healthcare). GST tag was removed by treatment with 100 μg of prescission protease/mg of fusion protein at 4°C overnight. Prescission protease was inactivated at 70°C for 15 min. The supernatant was concentrated to 3 mg/ml in storage buffer 15 mM Tris-HCl (pH 8.0), 150 mM NaCl, 1 mM dithiothreitol, and 10% glycerol. Wild type aKMT4 and its transferase deficient aKMT4-G38R proteins were purified as described previously (Niu et al., 2013).

### Protein Methylation Reactions

Standard protein methylation reactions were performed at 50°C as described as previously with minor modifications (Niu et al., 2013). The standard molar ratio of enzyme (aKMT4 methyltransferase) and substrate (MCM protein) is 3:1 unless otherwise specifically indicated. S-adenosyl-methionine (AdoMet) was used as a methyl donor. ^3^H-AdoMet (15 Ci/mmol, PerkinElmer Life Sciences) was used to detect tritium incorporation by autoradiography. MCM proteins from the methylation reactions were separated by 10% SDS-PAGE and transferred to PVDF membrane. The membrane was stained by Coomassie Brilliant Blue R250. ^3^H-methyl incorporation was detected by autoradiography on a high speed gadolinium oxysulfide intensifying screen.

### Antibodies

Polyclonal antibodies specific to MCM were raised and affinity-purified using recombinant GST-MCM or protein G resin.

### Immunoprecipitation

To detect the modifications of MCM *in vivo*, native MCM protein was prepared by an immunoprecipitation procedure. Lysates of 2 × 10^9^ *Sulfolobus* cells were added to anti-MCM coupled protein G beads. After extensive washes to remove non-specific binding proteins, 30 μl of 2x SDS loading buffer was added to the beads. Samples were boiled for 5 min and resolved by 10% SDS-PAGE.

### Mass Spectrometry

The methylated sites of the native MCM or recombinant MCM catalyzed *in vitro* by aKMT4 were determined by MS/MS. The native MCM or *in vitro* methylated recombinant MCM bands were sliced and in-gel digested with trypsin as described previously (Niu et al., 2013). To minimize the artificial *in vitro* methylation due to low sequence specificity of aKMT4 enzyme, an equimolar mixture of aKMT4 and MCM protein was used. Tryptic fragments were analyzed by Orbitrap Velos (Thermo Scientific). MS/MS analysis was performed on three biological replicates to obtain the final list of methylation sites.

### Helicase Assays

For the helicase assay, a fork-shaped DNA including a 35-nt single-stranded tail and a 44-nt duplex region was used as the substrate (Brewster et al., 2008). Sequences of the oligonucleotides S1 and S2 are listed in Supplementary Table S1. S2 was labeled with 100 μCi of [γ-^32^P]-ATP (Perkin Elmer Life Sciences, 3,000 Ci mmol^-1^) using T4 polynucleotide kinase. The labeled substrate was annealed to a complementary oligonucleotide (see Supplementary Table S1) at a molar ratio of 1:1.5 in 20 mM Tris-HCl, pH 8.0, and 100 mM NaCl and subsequently purified by standard PAGE procedure. Helicase assay was performed at 65°C for 1 h in a 10 μl volume containing 0.5 nM fork-shaped DNA, 200 nM (as monomer) of the indicated protein, 5 mM ATP in the buffer [30 mM Tris acetate (pH 8.0), 75 mM NaCl, 50 mM potassium acetate, 10 mM magnesium acetate and 1 mM dithiothreitol]. The reaction was terminated by adding 5 μl of stop solution containing 100 mM EDTA, 0.5% SDS, 0.1% bromophenol blue and 50% glycerol. Aliquots were then loaded on a 10% polyacrylamide gel in 1xTBE. Gels were running in 0.5xTBE plus 0.1% SDS for 25 min at 200 V before drying and autoradiography. The unwound DNA was detected by autoradiography and quantified by Quantity One (Bio-Rad Laboratories, Inc.). Helicase activity is calculated as the percentage of unwound DNA among total DNA substrates. Relative activity is defined as the ratio to the activity of untreated wild type MCM protein, which is normalized as 100%. Activity data represent the average of at least three independent experiments with standard deviations indicated by error bars.

### ATPase Assay

ATPase assay was performed in a 10 μl reaction volume containing 30 mM Tris acetate (pH 8.0), 75 mM NaCl, 50 mM potassium acetate, 10 mM magnesium acetate, 1 mM dithiothreitol, 1 mM [γ-^32^P]ATP (Perkin Elmer Life Sciences, 0.2 Ci/mmol) and 100 nM protein (Brewster et al., 2010). After incubation at 65°C for 30 min, the reaction was quenched by addition of 1 μl 0.5 M EDTA. A 1 μl aliquot from each reaction was applied to a pre-washed PEI cellulose TLC plate (Sigma–Aldrich), dried, and run for 3 h in 2 M acetic acid and 0.5 M LiCl buffer. Plates were dried before autoradiography and quantified by Quantity One (Bio-Rad Laboratories, Inc.). Relative ATPase activity is defined as the ratio to the ATPase activity of untreated wild type MCM, which is normalized as 100%.

### Electrophoretic Mobility Shift Assay (EMSA)

Electrophoretic mobility shift assay was performed in a 10 μl reaction volume containing 30 mM Tris acetate (pH 8.0), 75 mM NaCl, 50 mM potassium acetate, 10 mM magnesium acetate, 1 mM dithiothreitol, 10% glycerol, 0.5 nM fork-shaped DNA. Then reactions were immediately loaded on a 6% polyacrylamide gel in 1xTBE. Gels were running in 0.5xTBE for 50 min at 120 V before drying and autoradiography (Slaymaker et al., 2013).

### Protein Sequence Alignment and Structural Analysis

The amino acid sequence of MCM homologs was retrieved from all crenarchaeal organisms and aligned using MUSCLE v3.8.31 with default settings (Edgar, 2004). The surface hydrophobicity of the peptides containing the methylated sites was computed in ProtParam on the ExPASy Server (Wilkins et al., 1999). The methylated lysine residues were mapped to the three dimensional crystal structure of the near full length *S. solfataricus* MCM (ssoMCM; PDB ID 4FDG) that shares nearly identical amino acid sequence with sisMCM (Brewster et al., 2008; Slaymaker et al., 2013).

### Limited Tryptic Digestion

Limited tryptic digestion was performed by incubating the indicated samples (each sample contains 0.2 μg sisMCM) with 0.01 μg Trypsin (New England Biolabs) in the presence or absence of 1 mM ATP or its analog at 25°C for 15 min (Meyer et al., 2003). The reactions were terminated by adding 4xSDS-PAGE loading buffer. Digested protein was immediately separated by a 12% SDS-PAGE gel prior to silver staining (Thermo scientific, PageSilver^TM^ Silver Staining Kit).

### Differential Scanning Calorimetry

After standard *in vitro* methylation reaction in the presence of either wild type aKMT4 or aKMT4-G38R mutant enzyme, protein samples were loaded into the sample cell of a Nano-DSC II System (GE Healthcare; Johnson, 2013). Same volume of reaction buffer (400 μl) was loaded into the reference cell. Samples were scanned from 20 to 110°C at a rate of 2°C/min. Background value was determined using buffer only. Purified MCM protein or aKMT4 alone was also performed as controls. Origin software (Microcal) was applied for curve fitting and data analysis. Each result represents the average from at least three independent experimental repeats.

## Author Contributions

HL, XSC, YX, and QC conceived and designed research; YX, YN, JC, and QC performed the experiments; YF, XSC, and YX carried out structural and computational analysis; HL, QC, XSC, and YX analyzed the data and wrote the paper.

## Conflict of Interest Statement

The authors declare that the research was conducted in the absence of any commercial or financial relationships that could be construed as a potential conflict of interest.

## References

[B1] Ambler R. Rees M. (1959). Epsilon-N-Methyl-lysine in bacterial flagellar protein. *Nature* 184 56–57. 10.1038/184056b0 13793118

[B2] Azkargorta M. Wojtas M. N. Abrescia N. G. A. Elortza F. (2014). Lysine Methylation mapping of crenarchaeal DNA-Directed RNA polymerases by collision-induced and electron-transfer dissociation mass spectrometry. *J. Proteome Res.* 13 2637–2648. 10.1021/pr500084p 24625205

[B3] Barry E. R. Bell S. D. (2006). DNA replication in the archaea. *Microbiol. Mol. Biol. Rev.* 70 876–887. 10.1128/MMBR.00029-06 17158702 PMC1698513

[B4] Barry E. R. Lovett J. E. Costa A. Lea S. M. Bell S. D. (2009). Intersubunit allosteric communication mediated by a conserved loop in the MCM helicase. *Proc. Natl. Acad. Sci. U.S.A.* 106 1051–1056. 10.1073/pnas.0809192106 19164574 PMC2633543

[B5] Barry E. R. Mcgeoch A. T. Kelman Z. Bell S. D. (2007). Archaeal MCM has separable processivity, substrate choice and helicase domains. *Nucleic Acids Res.* 35 988–998. 10.1093/nar/gkl1117 17259218 PMC1807962

[B6] Beattie T. R. Bell S. D. (2011). Molecular machines in archaeal DNA replication. *Curr. Opin. Chem. Biol.* 15 614–619. 10.1016/j.cbpa.2011.07.017 21852183

[B7] Bochman M. L. Schwacha A. (2008). The Mcm2-7 complex has in vitro helicase activity. *Mol. Cell* 31 287–293. 10.1016/j.molcel.2008.05.020 18657510

[B8] Bochman M. L. Schwacha A. (2009). The Mcm Complex: unwinding the mechanism of a replicative helicase. *Microbiol. Mol. Biol. Rev.* 73 652–683. 10.1128/MMBR.00019-09 19946136 PMC2786579

[B9] Botting C. H. Talbot P. Paytubi S. White M. F. (2010). Extensive lysine methylation in hyperthermophilic crenarchaea: potential implications for protein stability and recombinant enzymes. *Archaea* 2010 106341 10.1155/2010/106341 PMC2929605 20811616

[B10] Brewster A. Slaymaker I. Afif S. Chen X. (2010). Mutational analysis of an archaeal minichromosome maintenance protein exterior hairpin reveals critical residues for helicase activity and DNA binding. *BMC Mol. Biol.* 11:62 10.1186/1471-2199-11-62 PMC2933578 20716382

[B11] Brewster A. S. Wang G. Yu X. Greenleaf W. B. Carazo J. M. Tjajadi M. (2008). Crystal structure of a near-full-length archaeal MCM: functional insights for an AAA+ hexameric helicase. *Proc. Natl. Acad. Sci. U.S.A.* 105 20191–20196. 10.1073/pnas.0808037105 19073923 PMC2629282

[B12] Brock T. D. Brock K. M. Belly R. T. Weiss R. L. (1972). Sulfolobus: a new genus of sulfur-oxidizing bacteria living at low pH and high temperature. *Arch. Microbiol.* 84 54–68. 10.1007/BF00408082 4559703

[B13] Choli T. Henning P. Wittmann-Liebold B. Reinhardt R. (1988). Isolation, characterization and microsequence analysis of a small basic methylated DNA-binding protein from the Archaebacterium, Sulfolobus solfataricus. *Biochim. Biophys. Acta Gene Struct. Exp.* 950 193–203. 10.1016/0167-4781(88)90011-5 3132977

[B14] Chong J. P. J. Hayashi M. K. Simon M. N. Xu R.-M. Stillman B. (2000). A double-hexamer archaeal minichromosome maintenance protein is an ATP-dependent DNA helicase. *Proc. Natl. Acad. Sci. U.S.A.* 97 1530–1535. 10.1073/pnas.030539597 10677495 PMC26469

[B15] Chu Y. Zhang Z. Wang Q. Luo Y. Huang L. (2012). Identification and characterization of a highly conserved crenarchaeal protein lysine methyltransferase with broad substrate specificity. *J. Bacteriol.* 194 6917–6926. 10.1128/JB.01535-12 23086207 PMC3510558

[B16] Chuikov S. Kurash J. K. Wilson J. R. Xiao B. Justin N. Ivanov G. S. (2004). Regulation of p53 activity through lysine methylation. *Nature* 432 353–360. 10.1038/nature03117 15525938

[B17] Clarke S. G. (2013). Protein methylation at the surface and buried deep: thinking outside the histone box. *Trends Biochem. Sci.* 38 243–252. 10.1016/j.tibs.2013.02.004 23490039 PMC3634909

[B18] Contursi P. Jensen S. Aucelli T. Rossi M. Bartolucci S. She Q. (2006). Characterization of the Sulfolobus host–SSV2 virus interaction. *Extremophiles* 10 615–627. 10.1007/s00792-006-0017-2 16896526

[B19] Edgar R. C. (2004). MUSCLE: multiple sequence alignment with high accuracy and high throughput. *Nucleic Acids Res.* 32 1792–1797. 10.1093/nar/gkh340 15034147 PMC390337

[B20] Egorova K. S. Olenkina O. M. Olenina L. V. (2010). Lysine methylation of nonhistone proteins is a way to regulate their stability and function. *Biochemistry (Mosc.)* 75 535–548. 10.1134/S0006297910050019 20632931

[B21] Erce M. A. Pang C. N. I. Hart-Smith G. Wilkins M. R. (2012). The methylproteome and the intracellular methylation network. *Proteomics* 12 564–586. 10.1002/pmic.201100397 22246820

[B22] Erzberger J. P. Berger J. M. (2006). Evolutionary relationships and structural mechanisms of AAA+ proteins. *Annu. Rev. Biophys. Biomol. Struct.* 35 93–114. 10.1146/annurev.biophys.35.040405.101933 16689629

[B23] Febbraio F. Andolfo A. Tanfani F. Briante R. Gentile F. Formisano S. (2004). Thermal stability and aggregation of sulfolobus solfataricus β-glycosidase are dependent upon the n-∊-methylation of specific lysyl residues: critical role of in vivo post-translational modifications. *J. Biol. Chem.* 279 10185–10194. 10.1074/jbc.M308520200 14660666

[B24] Fletcher R. J. Bishop B. E. Leon R. P. Sclafani R. A. Ogata C. M. Chen X. S. (2003). The structure and function of MCM from archaeal M. Thermoautotrophicum. *Nat. Struct. Mol. Biol.* 10 160–167. 10.1038/nsb893 12548282

[B25] Fletcher R. J. Shen J. Gómez-Llorente Y. Martín C. S. Carazo J. M. Chen X. S. (2005). Double hexamer disruption and biochemical activities of *Methanobacterium thermoautotrophicum* MCM. *J. Biol. Chem.* 280 42405–42410. 10.1074/jbc.M509773200 16221679

[B26] Hughes E. Burke R. M. Doig A. J. (2000). Inhibition of Toxicity in the β-Amyloid Peptide Fragment β-(25–35) Using N-Methylated Derivatives: a general strategy to prevent amyloid formation. *J. Biol. Chem.* 275 25109–25115. 10.1074/jbc.M003554200 10825171

[B27] Jenkinson E. R. Chong J. P. J. (2006). Minichromosome maintenance helicase activity is controlled by N- and C-terminal motifs and requires the ATPase domain helix-2 insert. *Proc. Natl. Acad. Sci. U.S.A.* 103 7613–7618. 10.1073/pnas.0509297103 16679413 PMC1472493

[B28] Jenkinson E. R. Costa A. Leech A. P. Patwardhan A. Onesti S. Chong J. P. J. (2009). Mutations in subdomain b of the minichromosome maintenance (mcm) helicase affect dna binding and modulate conformational transitions. *J. Biol. Chem.* 284 5654–5661. 10.1074/jbc.M806973200 19116205 PMC2683335

[B29] Johnson C. M. (2013). Differential scanning calorimetry as a tool for protein folding and stability. *Arch. Biochem. Biophys.* 531 100–109. 10.1016/j.abb.2012.09.008 23022410

[B30] Kelman L. M. Kelman Z. (2014). Archaeal DNA Replication. *Annu. Rev. Genet.* 48 71–97. 10.1146/annurev-genet-120213-092148 25421597

[B31] Kelman Z. Lee J.-K. Hurwitz J. (1999). The single minichromosome maintenance protein of *Methanobacterium thermoautotrophicum* ΔH contains DNA helicase activity. *Proc. Natl. Acad. Sci. U.S.A.* 96 14783–14788. 10.1073/pnas.96.26.14783 10611290 PMC24725

[B32] Kim Y. Quartey P. Li H. Volkart L. Hatzos C. Chang C. (2008). Large-scale evaluation of protein reductive methylation for improving protein crystallization. *Nat. Methods* 5 853–854. 10.1038/nmeth1008-853 18825126 PMC2678869

[B33] Martin C. Zhang Y. (2005). The diverse functions of histone lysine methylation. *Nat. Rev. Mol. Cell Biol.* 6 838–849. 10.1038/nrm1761 16261189

[B34] McGeoch A. T. Trakselis M. A. Laskey R. A. Bell S. D. (2005). Organization of the archaeal MCM complex on DNA and implications for the helicase mechanism. *Nat. Struct. Mol. Biol.* 12 756–762. 10.1038/nsmb974 16116441

[B35] Meyer A. Gillespie J. Walther D. Millet I. Doniach S. Frydman J. (2003). Closing the folding chamber of the eukaryotic chaperonin requires the transition state of ATP hydrolysis. *Cell* 113 369–381. 10.1016/S0092-8674(03)00307-6 12732144

[B36] Moreau M. J. Mcgeoch A. T. Lowe A. R. Itzhaki L. S. Bell S. D. (2007). ATPase site architecture and helicase mechanism of an archaeal MCM. *Mol. Cell* 28 304–314. 10.1016/j.molcel.2007.08.013 17964268

[B37] Niu Y. Xia Y. Wang S. Li J. Niu C. Li X. (2013). A prototypic lysine methyltransferase 4 from archaea with degenerate sequence specificity methylates chromatin proteins Sul7d and Cren7 in different patterns. *J. Biol. Chem.* 288 13728–13740. 10.1074/jbc.M113.452979 23530048 PMC3650410

[B38] Onesti S. MacNeill S. (2013). Structure and evolutionary origins of the CMG complex. *Chromosoma* 122 47–53. 10.1007/s00412-013-0397-x 23412083

[B39] Polevoda B. Sherman F. (2007). Methylation of proteins involved in translation. *Mol. Microbiol.* 65 590–606. 10.1111/j.1365-2958.2007.05831.x 17610498

[B40] Pucci B. De Felice M. Rocco M. Esposito F. De Falco M. Esposito L. (2007). Modular organization of the sulfolobus solfataricus mini-chromosome maintenance protein. *J. Biol. Chem.* 282 12574–12582. 10.1074/jbc.M610953200 17337732

[B41] Pucci B. De Felice M. Rossi M. Onesti S. Pisani F. M. (2004). Amino acids of the *Sulfolobus solfataricus* mini-chromosome maintenance-like DNA helicase involved in DNA binding/remodeling. *J. Biol. Chem.* 279 49222–49228. 10.1074/jbc.M408967200 15371413

[B42] Randell J. C. W. Fan A. Chan C. Francis L. I. Heller R. C. Galani K. (2010). Mec1 is one of multiple kinases that prime the mcm2-7 helicase for phosphorylation by Cdc7. *Mol. Cell* 40 353–363. 10.1016/j.molcel.2010.10.017 21070963 PMC3021128

[B43] Sakakibara N. Kelman L. M. Kelman Z. (2009). Unwinding the structure and function of the archaeal MCM helicase. *Mol. Microbiol.* 72 286–296. 10.1111/j.1365-2958.2009.06663.x 19415794

[B44] Sheu Y.-J. Stillman B. (2006). Cdc7-Dbf4 phosphorylates MCM proteins via a docking site-mediated mechanism to promote s phase progression. *Mol. Cell.* 24 101–113. 10.1016/j.molcel.2006.07.033 17018296 PMC2923825

[B45] Slaymaker I. Chen X. (2012). “MCM structure and mechanics: what we have learned from archaeal MCM,” in *The Eukaryotic Replisome: a Guide to Protein Structure and Function*, ed. Macneill S. (Berlin: Springer), 89–111. 10.1007/978-94-007-4572-8_6 22918582

[B46] Slaymaker I. M. Fu Y. Toso D. B. Ranatunga N. Brewster A. Forsburg S. L. (2013). Mini-chromosome maintenance complexes form a filament to remodel DNA structure and topology. *Nucleic Acids Res.* 41 3446–3456. 10.1093/nar/gkt022 23361460 PMC3597688

[B47] Tye B. K. (1999). MCM proteins in DNA replication. *Annu. Rev. Biochem.* 68 649–686. 10.1146/annurev.biochem.68.1.649 10872463

[B48] Vieille C. Zeikus G. J. (2001). Hyperthermophilic enzymes: sources, uses, and molecular mechanisms for thermostability. *Microbiol. Mol. Biol. Rev.* 65 1–43. 10.1128/MMBR.65.1.1-43.2001 11238984 PMC99017

[B49] Wilkins M. R. Bairoch A. Sanchez J. C. Williams K. L. Appel R. D. Hochstrasser D. (1999). Protein identification and analysis tools in the ExPASy server. *Methods Mol. Biol.* 112 531–532. 10027275 10.1385/1-59259-584-7:531

[B50] Wu S. C. Zhang Y. (2009). Minireview: role of protein methylation and demethylation in nuclear hormone signaling. *Mol. Endocrinol.* 23 1323–1334. 10.1210/me.2009-0131 19407220 PMC2737564

[B51] Zhang X. Huang Y. Shi X. (2015). Emerging roles of lysine methylation on non-histone proteins. *Cell. Mol. Life Sci.* 72 4257–4272. 26227335 10.1007/s00018-015-2001-4 PMC11114002

[B52] Zhang X. Wen H. Shi X. (2012). Lysine methylation: beyond histones. *Acta Biochim. Biophys. Sin.* 44 14–27. 10.1093/abbs/gmr100 22194010

