# Supplementary material for: The Helicase Activity of Hyperthermophilic Archaeal MCM is Enhanced at High Temperatures by Lysine Methylation
Source: Front Microbiol. 2015 Nov 9;6:1247. doi: 10.3389/fmicb.2015.01247 (PMC4639711; doi:10.3389/fmicb.2015.01247)
Supplement: Supplementary file 1 [file Table_1.PDF]

SUPPLEMENTARY MATERIAL

Supplementary Table S1 and Figures S1-S2

The helicase activity of hyperthermophilic archaeal MCM is enhanced at high temperatures by lysine methylation

Yisui Xia[1], Yanling Niu[1], Jiamin Cui[1], Yang Fu[2,3,4], Xiaojiang S. Chen[2,3,4], Huiqiang Lou[1*], and Qinhong Cao[1*]

*To whom correspondence should be addressed. Tel/Fax: 8610-62734504; E-mail: lou@cau.edu.cn. Correspondence may also be addressed to: Qinhong Cao, Tel/Fax: 8610-62734304, E-mail: caoqinhong@cau.edu.cn

Supplementary Table S1. Oligonucleotides used in this study.

Primer Name	Primer Squence	Usage
MCM-F	GTTCCGCGTGGATCCCCGATGGAAATTCCTAGCAAACA	Cloning MCM in pGEX-6P-1
MCM-R	TCAGTCACGATGCGGCCGCTTAGACTTTTTTGTAACATTC	
sisMCM-K280281M-F	GAAGTAACAATCTCTGAAGAAGATGAGATGATGATCAAGGACTTA	Site-directed mutagenesis of MCM
sisMCM-K280281M-R	CCAAGGATCCTTAGCTAAGTCCTTGATCATCATCTCATCTTCTTC	
sisMCM-K545546M-F	ATTACAGACTTCTTCGTGGAGATGAGGATGATGAGTTCGGAAACT	Site-directed mutagenesis of MCM
sisMCM-K545546M-R	TATTGGGCTATCGGGAGTTTCCGAACTCATCATCCTCATCTCCAC	
sisMCM-K650L-F	GAGTGTGCAAAGGTTAAGGATATACTACTAGAAGCACAACAAGTT	Site-directed mutagenesis of MCM
sisMCM-K650L-R	ACTTTTTTCGATACCAACTTGTTGTGCTTCTAGTAGTATATCCTT	
S1	$(dT)_{35}$GCTCGTGCAG-ACGTCGAGGTGAGGACGAGCTCCTCGTGACCACG	dsDNA substrate for helicase and EMSA
S2	CGTGGTCACGAGGAGCTCGTCCTCACCTCG-ACGTCTGCACGAGC$(dT)_{35}$	

Supplementary Figure S1.

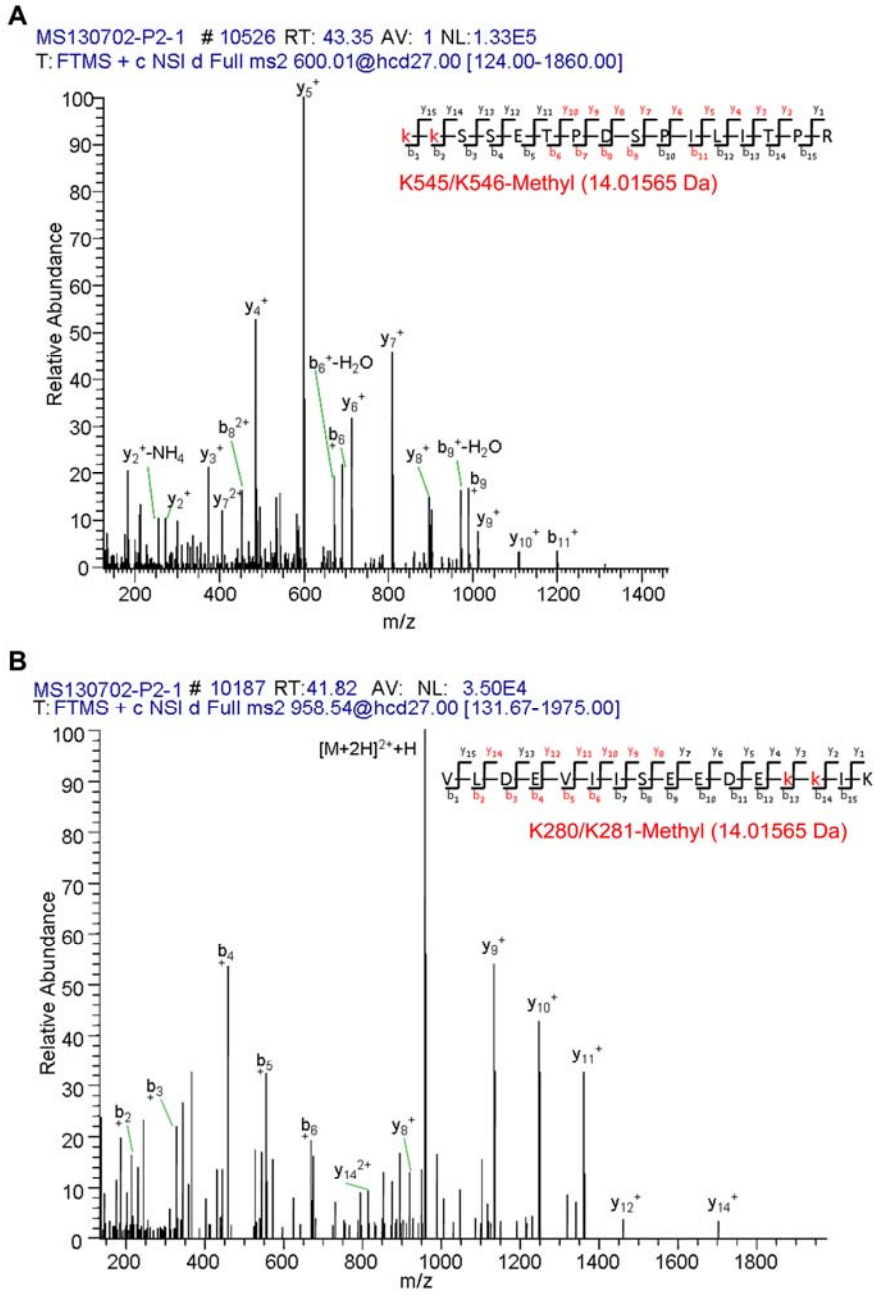

Figure S1 (Related to Figure 1). Representative spectra of MCM peptide containing K545/K546me (A) and K280/K281me (B). “b” and “y” ions with *m/z* difference by 14 may contain a mono-methylated lysine. The MS/MS analysis was conducted as described in Figure 1C.

Supplementary Figure S2.

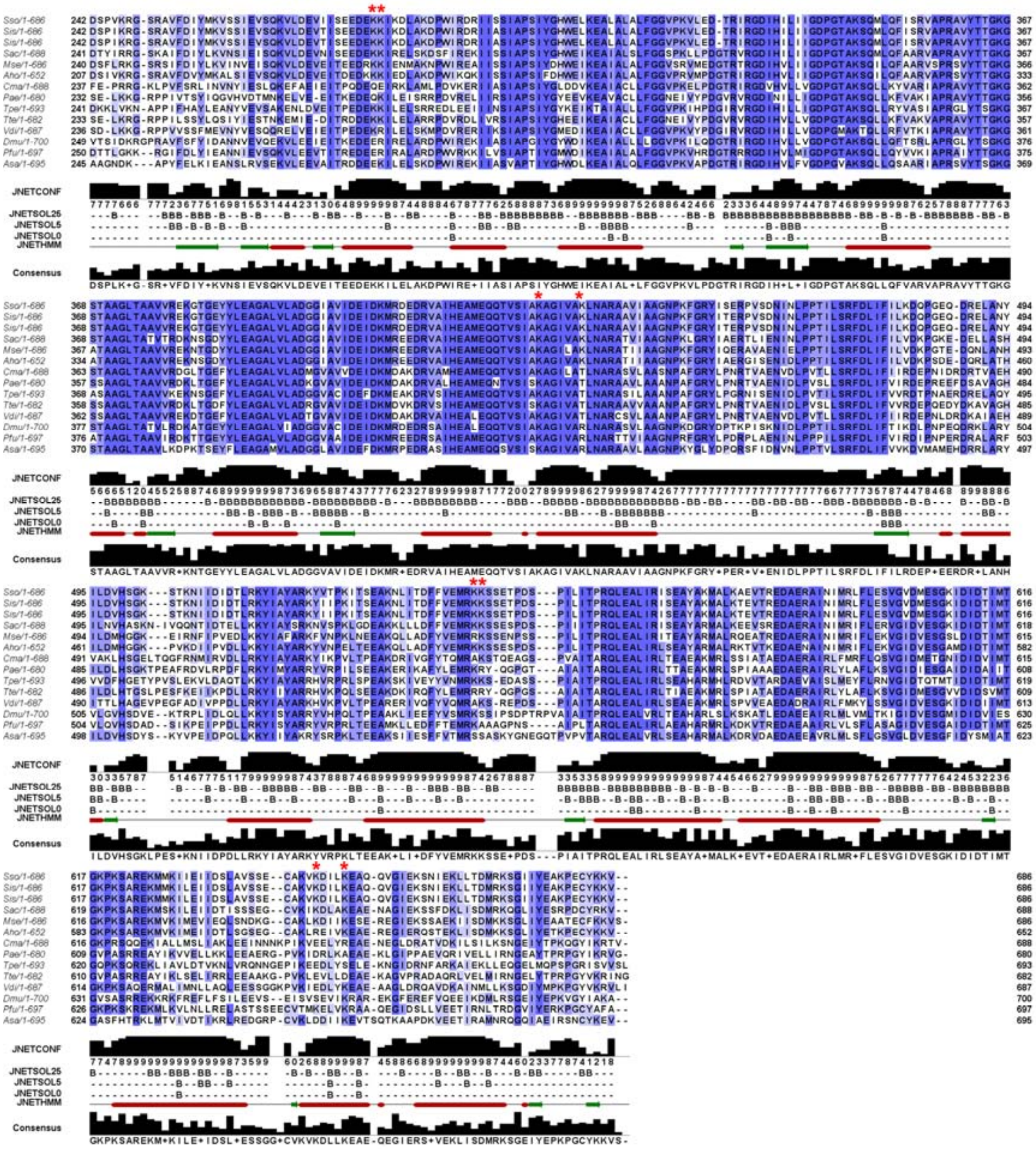

Figure S2 (Related to Figure 1). MCM methylation may be conserved among crenarchaeota. Amino acid sequences of MCM from representative crenarchaea species were aligned. The most conserved amino acids were shaded in blue. Methylated lysine residues identified in MS/MS spectra were marked with a red asterisk (*).
